# Utilization of Function Generation Synthesis on Biomimetics: A Case Study on Moray Eel Double Jaw Design

**DOI:** 10.3390/biomimetics7040145

**Published:** 2022-09-28

**Authors:** Mertcan Koçak, Mustafa Volkan Yazıcı, Efecan Akdal, Fatih Cemal Can, Erkin Gezgin

**Affiliations:** 1 Department of Mechatronics Engineering, İzmir Katip Çelebi University, 35620 İzmir, Turkey; 2 Department of Mechanical Engineering, İzmir Katip Çelebi University, 35620 İzmir, Turkey; 3 Department of Mechanical Engineering, Yaşar University, 35100 İzmir, Turkey

**Keywords:** biomimetics, moray eel, function generation synthesis, multiple synthesis, optimization

## Abstract

Throughout history, humans have observed living or non-living things in nature and then imitated them in relation to these observations. This is due to the fact that the energy found in nature is generally consumed at an optimal level in order for it to endure. Biomimetic inspiration in many designs and applications is widely displayed, including within the field of engineering. In this paper, we were inspired by the double set of jaws found in the moray eel, which gives this fish a huge advantage while hunting, with a mobile pharyngeal jaw that works together with its oral jaw in order to overcome ineffective suction capabilities. A procedure that mimics the hunting motion of the moray eel was utilized by considering the overall movement as a single degree of freedom with multiple outputs on account of the repeating motion that is required during hunting. This procedure includes structural and dimensioning synthesis, wherein the latter was utilized with analytic kinematic synthesis for each linkage transfer. The flexibilities in parameters were taken into account with a novel multiple iterative kinematic synthesis algorithm that resulted in various mechanisms with the same purpose. Among the excessive number of resultant mechanisms, the optimization was carried out by considering the highest torque transmission ratio at critical timings that were specified as bio-constraints. In the end, the kinematic movement validation was utilized.

## 1. Introduction

Scientists have been trying to understand biological systems from various points of view throughout history. From an engineering point of view, the mechanical and electrical analogies of biological systems in technical works are said to have been started, at least, as long ago as Leonardo Da Vinci, Galvani, and Volta. The term “biomimetic” was born out of a need for those within technical fields to be able to describe the combination of some areas of biology with that of science or engineering wherein this combination wished to mimic the different functionalities of living or non-living creatures in the environment, including humans, with advances in material science, technology, and an understanding of biological systems [1]. Throughout these advances, the designing processes of many biomimetic machines that benefit humans have been inspired by nature [2,3]. It is undeniable that things in nature tend to use their energy at an optimum in order to endure. Thus, there is a duality between engineering and nature, as the performance of biologically inspired robots has robustness and sustainability [4]. Scientists have recently introduced this design approach of combining bio-inspiration to engineering students by adding it to curriculums as a new way to increase technical challenges [4]. Engineering companies should also implement biomimetic design processes into their procedures by changing organizational structures that, in the end, may combine the fields of engineering and biology together [5]. Biomimetic robots are robots that imitate not only humans but also animals that will eventually adapt to the environment so that they can learn and react faster, while, at the same time, may have novel mechanisms and manipulator structures according to their needs [6,7]. Over the past decade, many robotic devices, whose designs are based upon certain biological species in terms of user functionality in practical applications, have been developed, such as: humanoid robots [8,9] snake-like robots [10,11], swimming robots [12,13,14], flying robots [15,16], and even plants [17]. Moreover, there are studies where the functionality of certain parts of a biological species are mimicked without considering appearance, such as [18] and Liu et al.’s study [19] which is based on the compliant spine mechanism of the leaf-feeding insect, Motschulsky. In this study, they designed a wheeled wall-climbing robot and analyzed its performance with experiments and, eventually, concluded that climbing performance was improved by the bio-inspired compliant spine mechanism.

In this paper, the generated motion of the proposed mechanism was inspired by the combination of two different sets of jaws that have evolved in some fishes. While one of these jaws is the oral jaw that collects food, the other is the pharyngeal jaw normally located in the pharynx of the fish that transports the food into the esophagus [20,21]. Among these fishes that have this kind of jaw, moray eels have certain advantages in terms of hunting with respect to the point of view of this study. Although moray eels do not have effective suction feeding abilities, this disadvantage is overcome by a well-developed and well-positioned pharyngeal jaw. More so than other fishes with a pharyngeal jaw, these creatures are capable of pulling prey into the esophagus with the help of their additional jaw, which gives them a huge advantage during hunting. Mehta and Wainwright [22] observed the moray eels by using high-speed video in a feeding sequence and they observed that the pharyngeal jaws are used together with the oral jaw. Moreover, it was noted that the upper and lower teeth are very convenient for extreme prey transport conditions during retraction and protraction. The advantage of this system overall appears especially when large prey is the target. In [23], Triyonoputro et al. designed and manufactured a double-jaw hand mechanism that mimicked the functionality of the moray eel jaw system. They imitated three motions of the mobile pharyngeal jaw: opening and moving forward, biting the prey, and pulling the prey with the double jaw and with their outer and inner gripper design. This overall hand system has four degrees of freedom (DOF) in total, with one of these dedicated to making the two grippers mobile.

When considering combining mechanisms with biomimetic studies, it is crucial to have knowledge about designing kinematic motions according to resultant tasks, which is, in this study, the motion of two sets of jaws dependently. Motion study of machines and mechanisms may be achieved by two methods of analysis and synthesis [16]. Simply, they can be described as studies that deal with mechanical systems in order to understand or fulfill certain motion characteristics. Kinematic synthesis is a design process for the functional dimensioning of mechanisms according to the required task, which allows the system DOF to be kept lower than the task degree of freedom [24]. The decision and procedures of the kinematic synthesis methods are the most important steps of the mechanical design procedure in terms of dimensioning the parts. Moreover, error characteristics of the designed mechanism are obtained by kinematic synthesis. Basically, there are three types of analytic kinematic synthesis methods used to designate the output characteristic of the mechanism, which are: function generation synthesis, body guidance synthesis, and path generation synthesis [25].

Function generation synthesis attempts to design the construction parameters of the mechanisms in order to provide a specified function relationship between the input and the output motion [26,27]. Path generation synthesis [28] guides a coupler point along a specified path and body guidance synthesis [29] is used when the body of the mechanism guides through a number of specified positions. Since these methodologies are based on keeping the DOF smaller than that of the tasks, it is not possible to obtain an exact solution, therefore, different approximation techniques are applied, and, eventually, motion is achieved with finite error. In applying this kind of approximation, precision points are defined regarding which motion is fulfilled. Overall, the number of the precision points must be equal to the number of the unknown construction parameters used in the synthesis [24].

In order to conclude a dimensioned mechanism or manipulator to serve as task-specific by using a function generation synthesis methodology, as mentioned above, it is a requirement to have an input–output relation in order to fulfill the motion. This relationship has quite the effect on the resultant mechanism. On the other hand, if the required output paths are predictable, at least for some precision points but not for the input motion or range, then it is possible to construct a multiple input–output relationship within the permitted range of inputs and corresponding outputs. With an excessive number of input–output ranges, multiple solutions should be handled. Advances in computing technologies allow them to be used in different areas, including optimization computing. Ouezdou et al. [30] used an iterative optimization technique in kinematic synthesis with a rigid body guidance problem with six DOF manipulators. The Distributed Optimization Algorithm (DOM) was used to obtain analytical expressions for the minimal value of each parameter, whereas the dimensional parameters of the manipulator were determined. Without considering the kinematic synthesis methodology, in Kivela’s thesis [31], a generic method for finding an optimal solution by using the Levenberg–Marguardt method was developed, eventually ranking and choosing the resultant robot according to the selected performance measure made possible. Patel and Sobh [32] developed a methodology to determine optimal manipulator configurations based on task descriptions. The idea behind this methodology was to get all of the possibilities in dimensioning that could reach the required poses, among which the best kinematic performance could be selected within the user-defined constraints with the least power consumption.

The purpose of this work is to design a mechanism that mimics the motion of two jaw sets of the moray eel with a single actuator. The oral jaw is driven by an inverted slider–crank mechanism that is responsible for initially catching the prey. The second set of the jaw (the pharyngeal jaw) pulls the prey inside through the esophagus, which is composed of two different mechanisms; anti parallel four-bar mechanism as the biting part and an offset slider–crank mechanism as the translating part between the throat and out end of the mouth. Analytic kinematic synthesis methodologies were applied in order to design a planar-type of mechanism by using an algorithm called a synthesis algorithm in order to have an iterative synthesis procedure that is made possible due to the flexibility of the parameters. Among different results, optimization is carried out in order to reach the most suitable mechanism to deliver the highest torque at a specified moment from the actuator to the outputs that are obtained with virtual work methods in each step. Finally, the mechanism is designed based on kinematics in order to validate the procedure.

## 2. Bio-Constraints and Structural Synthesis

**Figure 1:**
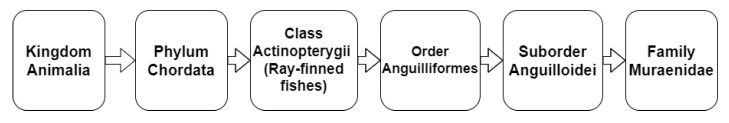
The moray eel’s biological classification tree.

The number of fishes that live in the ocean is a mystery. Eels are ray fin fish that belong to the Anguilliformes order, and moray eels (Muraenidae) are one of the members of this eel family. There are approximately 200 species of moray eel types in the world [33] and their heights can be 60–80 cm to 2–3 m [34]. In Figure 1, the moray eel’s biological classification tree [34] is shown.

**Figure 2:**
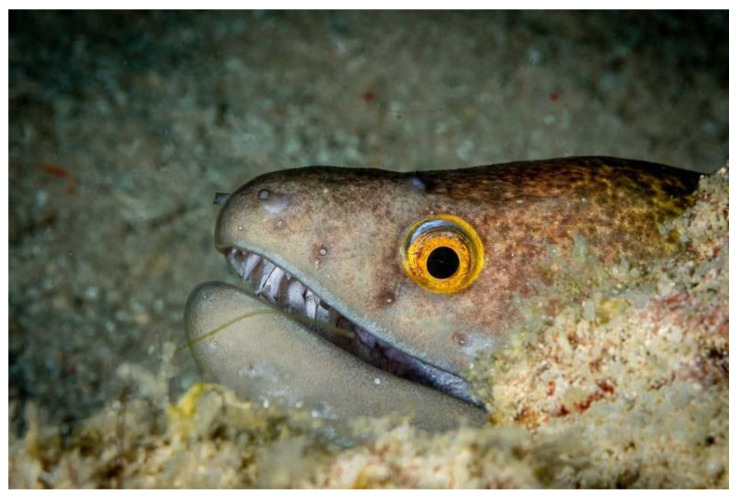
Moray eel. Permission for photo was taken from Yalçın Aydın.

The moray eels’ set of jaws is the main difference that makes this research team want to study them. These creatures have two separate jaws; an oral jaw and a pharyngeal jaw. None of the jaw sets have become blunt due to the fact that they give a huge advantage to those creatures under water and are very useful and frequently used during hunting (Figure 2).

**Figure 3:**
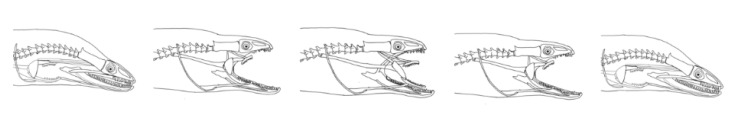
The approximate motion of the moray eel during hunting.

In terms of mechanism structures, the oral jaw can perform an opening and closing motion and the pharyngeal jaw can additionally slide inside the oral jaw as a whole apart from its opening and closing motion. The hunting motion of the moray eel’s overall jaw structure can be treated as a repeatable motion when the prey is being hunted. The approximate motion of the moray eel during hunting can be visualized as shown in Figure 3.

In the nature of the moray eel’s hunting, firstly, they squeeze their prey with their oral set of jaws and make the prey ineludible. Then, the pharyngeal jaw comes into action from the throat to the outside of the mouth in order to snatch the prey to pull it into the esophagus. Before pulling the prey into the esophagus, the oral jaw should release the prey in order for it to be pulled easily. Thus, the pharyngeal jaw of moray eel has a specific motion characteristic working together with the oral jaw, which is combined with both the translation to get out of the oral jaw and to pull back the jaw together with the prey to the esophagus, and the rotation to bite and squeeze the prey. In order to mimic the motion characteristics of the moray eel on a mechanism that is dependent on each other, bio-constraints that define different poses of the pharyngeal and the oral jaws to be fulfilled in terms of kinematics for this work can be visualized in Table 1.

**Figure 4:**
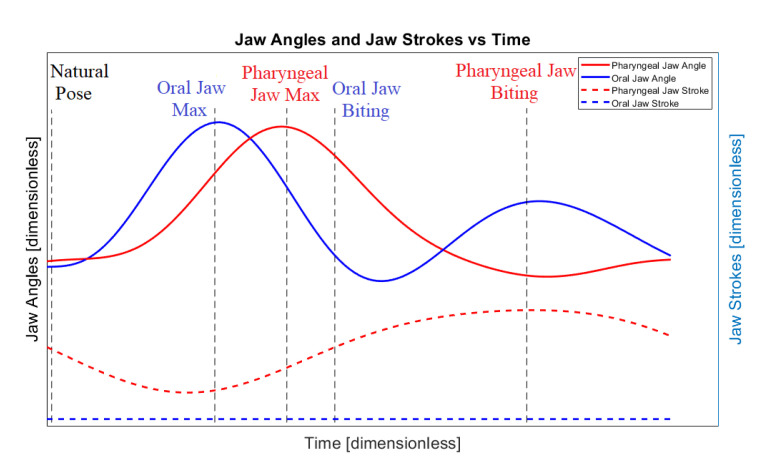
Approximation of the position and the angle requirements for both jaws.

By means of considerations in the hunting of the moray eel with a visualization in Table 1, the whole mechanism has specific position and angle requirements at each instant of time that can be approximated as shown in Figure 4.

The graphic in Figure 4 shows the rotational angle and translational stroke of the pharyngeal and oral jaws of the moray eel with respect to each other through the time. The solid red line is the angle of the pharyngeal jaw, the solid blue line is the oral jaw, the dashed red line is the stroke of the pharyngeal jaw, and the dashed blue line is the stroke of the oral jaw. In this work, the ultimate goal is specified with these approximate movement characteristics and can be treated as bio-constraints of the motion.

While the only rotational actuator that is placed in a proper point inside the biomimetic mechanism is driven with a constant rotational velocity, the required bio-constraints of the motions can be sorted as follows:

- If the pharyngeal jaw is at the deep in the throat, the pharyngeal jaw should be in an open position. At the same time, the oral jaw should be in an open position and ready for the biting action.
- If the pharyngeal jaw travels from the throat to out of the mouth, the pharyngeal jaw should be in an open position. At the same time, the oral jaw should be in a closed position when the prey is caught.
- If the pharyngeal jaw reaches out of the mouth, the pharyngeal jaw should be in a closed position (the action of biting). At the same time, the oral jaw should be in an opening position when the prey is just released.
- If the pharyngeal jaw travels from out of the mouth to the throat, the pharyngeal jaw should be in a closed position (carrying prey to the esophagus). At the same time, the oral jaw should be in a closing manner to the natural pose, which is a closed oral jaw.

Before the structural design of the mechanism, functional design constraints should also be defined, which have a huge impact on the chosen structure. As is seen from the biomimetic motion characteristics, the overall motion of both jaws is dependent on each other during hunting. By considering the whole motion of both jaws, the mechanism may be constructed with multiple degrees of freedom, however, depending on the motion characteristics during the motion, it is highly possible to face control difficulties. For the sake of simplicity, this overall motion can be reduced to a single DOF motion including many sets of mechanisms that are driven by a common source. This creates dependency, which results in task-based results, however, it can be adapted to other automation applications without considering any relative control difficulties. The increase in overall safety is also an advantage in such a design due to the motion constraints.

Avoiding an increase in mechanical complexity, another functional design constraint, defined as the common actuation source, is a rotary electric motor that drives the whole movement from an intersection point so that there will be no additional motion transferring other than the structural mechanisms. The moray eel’s overall hunting characteristics will be integrated without any change during the mechanism’s design.

Considering biomimetic and functional design constraints, the structure of the mechanism is chosen to be linkage type with different movement abilities in order to achieve multiple motions with kinematic synthesis methodology, since task mobility is greater than the targeted mobility.

**Figure 5:**
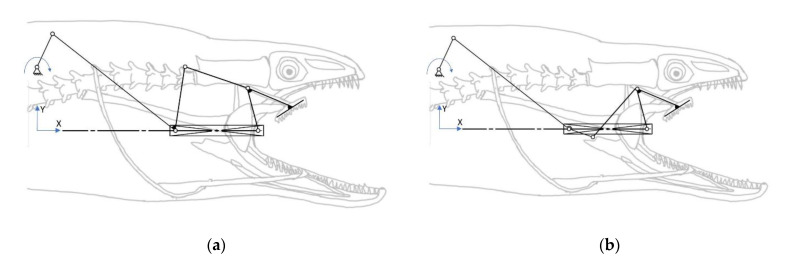
Kinematic chain representation of the pharyngeal jaw: slider–crank mechanism with (**a**) a four-bar mechanism; (**b**) an anti-parallel four-bar mechanism.

Considering the motion of the pharyngeal jaw that has both translation and rotation motion, the structure of the mechanism has to contain some asymmetry due to the bio-constraints. This asymmetry may be allowable by adding some offset to a slider–crank mechanism. For this reason, a mechanism that combines a slider–crank mechanism with an offset for sliding and a four-bar mechanism for rotating was chosen, with the possibility of two different types (Figure 5).

As seen in the kinematic chain representation of the structure, the actuation source of the movement is placed in the moray eel’s body in a grounded position. The coupler link of the slider–crank mechanism is actually the input source for the translating four-bar mechanism itself. By considering the bio-constraints of the overall design process, as the slider moves out of the throat (*+x* direction in Figure 5a), which is possible by rotating the input source in a clockwise direction, the output of the sliding four-bar mechanism should rotate in the direction of the biting (clockwise direction) in order to catch the prey. In the other direction of the slider mechanism (*-x* direction in Figure 5a), the output of the sliding four-bar mechanism should keep its position closed (biting) in order to not allow the prey to flee. As a result, the structure of the sliding four-bar mechanism should be changed to an anti-parallel type in order to achieve this movement constraint (Figure 5b). With this configuration, it is possible to achieve a closing action during the slider moves out of the mouth.

**Figure 6:**
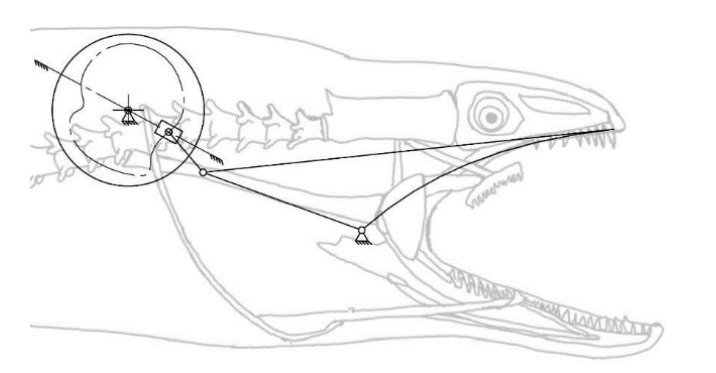
Kinematic chain representation of the oral jaw: inverted slider–crank mechanism.

For mechanical simplicity and to avoid motion transmission with additional linkages or belts, the motion source of the overall mechanism is placed in the common point where it is the final actuator location. Considering the motion of the oral jaw, it should only achieve the rotational motion when necessary. According to the bio-constraints, in a one-time cycle of a full rotation of the rotary actuator, the rotational motion of the oral jaw should be utilized two times, one of which is to catch the prey with the oral jaw and the other is to keep the position as a natural pose, which is visualized in Table 1. On the other hand, as the functional design constraint, the mechanism should be a single DOF. As a result, the mechanism chosen to achieve this overall motion is the inverted slider–crank mechanism, where the input is the prismatic joint as seen from the kinematic chain representation in Figure 6. However, since it was planned to have a single rotational actuator on the system to drive all of the motion, this prismatic joint motion as input is given to the system with a cam profile, whose profile is extracted according to the timings of the oral jaw.

**Figure 7:**
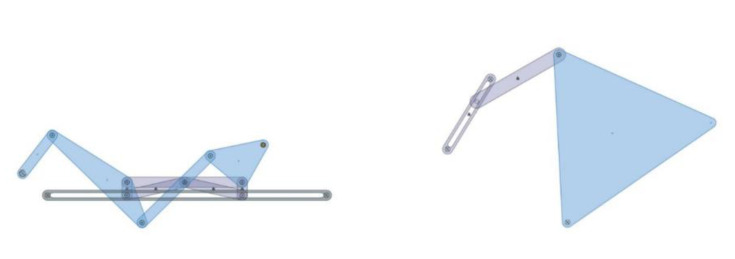
The structural design of the pharyngeal jaw and the oral jaw.

As a result, the structures of both jaw mechanisms are chosen as an offset slider–crank mechanism combined with an anti-parallel four-bar mechanism for the pharyngeal jaw, and an inverted slider–crank mechanism with the input of a prismatic joint that is driven with a cam profile (Figure 7).

**Figure 8:**
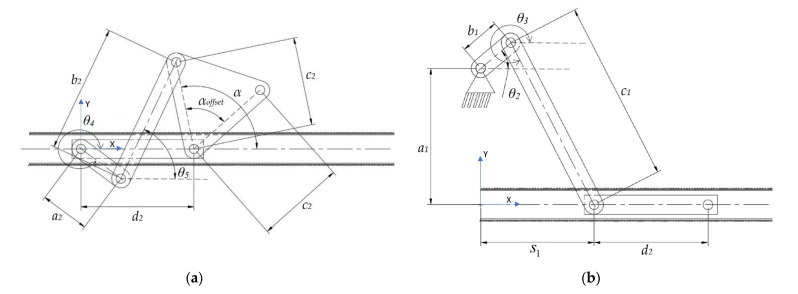
Pharyngeal jaw: (**a**) squeezing action mechanism; (**b**) translation action mechanism.

As a result of this structural synthesis, the squeezing action of the pharyngeal jaw can be achieved by a four-bar mechanism (Figure 8a), while the translation action is possible by constructing an offset slider–crank mechanism (Figure 8b).

**Figure 9:**
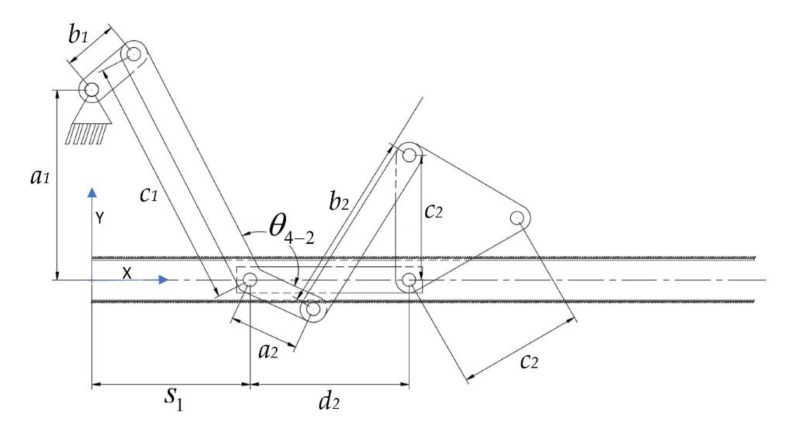
The combined structure of the pharyngeal jaw.

Due to the design goal, both mechanisms are combined together with the common actuator. As a result, the mechanical structure of the pharyngeal jaw is composed of one offset slider–crank mechanism and one anti-parallel four-bar mechanism that is placed on the slider and driven by it (Figure 9).

**Figure 10:**
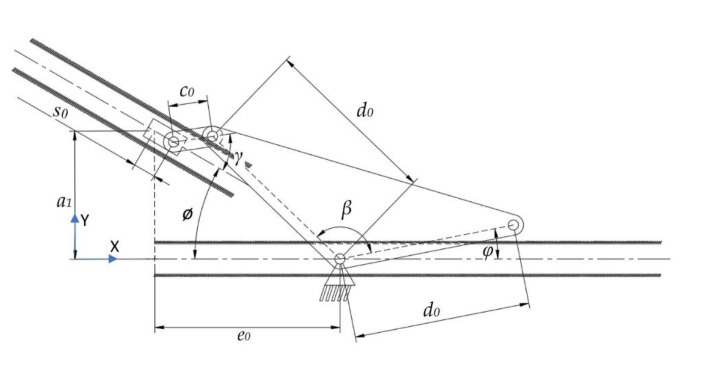
The combined structure of the oral jaw.

As in the structural synthesis part, the oral jaw is constructed as an inverted slider–crank mechanism and this mechanism with labeled parameters can be seen in Figure 10.

After the structural design is utilized, the dimensioning of the links comes into action as the next step. The functions that define the input–output relations should be revealed and the analytic kinematic synthesis procedure should be carried out.

## 3. Kinematic Synthesis

### 3.1. Kinematic Synthesis of the Pharyngeal Jaw

In the proposed design, the motion of the pharyngeal jaw is generated by the combination of two different mechanisms; the anti-parallel four-bar mechanism and the offset slider–crank mechanism. The main mechanism is formed by the serial connection of these two mechanisms. Input motion acts on the crank (*b*_1_) of the slider–crank mechanism, and, thus, translational motion is generated. The translational motion of the slider provides the translational motion of the four-bar mechanism which, in turn, provides the rotational motion of the pharyngeal jaw. To ensure the constraint that the overall mechanism is actuated by a single actuator, the rotational motion input of the four-bar mechanism is provided by one of the links of the slider mechanism. This situation is ensured by the rigid connection of the slider’s coupler link (*c*_1_) and the four-bar link (*a*_2_). Thus, the motion of the slider makes (*a*_2_) rotate anti-clockwise so that the translated jaw link (*c*_2_) tends to close the pharyngeal jaw (the biting action). In this regard, it was decided that the most suitable mechanism type utilizing kinematics and geometrical constraints for the rigid connection of these two links is the anti-parallel four-bar mechanism. In light of the given information, both mechanisms will be synthesized by analytical methods.

#### 3.1.1. Kinematic Synthesis of the Four-Bar Mechanism and the Pharyngeal Jaw

In Section 2, the motion profile of the pharyngeal jaw was explained in detail. The motion of this jaw can be examined as a combination of pure rotation and pure translation. In this section, the four-bar mechanism which presents pure rotation of this jaw will be examined.

The rotational motion of the pharyngeal jaw is constrained by the natural characteristics of the moray itself. In other words, output angle (*α*) and its time steps are bio-constraints (Figure 8a). In order to ensure these characteristics, a function must be described between the input angle (*θ*_4_), which comes from the slider, and the output angle (*α*). As explained before, such necessity can be solved by function generation synthesis and in this part of the study, this method is used.

In this configuration of the four-bar mechanism, the distance between the grounded joints is assumed as a biomimetic constraint and defined by the actual size of the slider of the pharyngeal jaw. This quantity affects the construction parameters (unknown link lengths of the four-bar mechanism) linearly. Therefore, it can be concluded that this parameter (*d*_2_ in Figure 8a) is a scale factor for the construction parameters and the pharyngeal jaw and it is a predefined and known parameter.

Function generation synthesis starts with the *x* and *y* components of closed-loop equations which can be visualized in Figure 8a and revealed in Equations (1) and (2):

(1) $$a_{2}\cos\theta_{4}+b_{2}\cos\theta_{5}=d_{2}+c_{2}\cos\alpha$$

(2) $$a_{2}\sin\theta_{4}+b_{2}\sin\theta_{5}=c_{2}\sin\alpha$$

In order to obtain a relationship between *α* and *θ*_4_, *θ*_5_ should be eliminated from the equations by adding the squares of Equations (3) and (4):

(3) $$b_{2}\cos\theta_{5}=d_{2}+c_{2}\cos\alpha -a_{2}\cos\theta_{4}$$

(4) $$b_{2}\sin\theta_{5}=c_{2}\sin\alpha -a_{2}\sin\theta_{4}$$

The resultant equation is shaped as follows to form the objective function as in Equation (5):

(5) $$\begin{array}{l} b_{2}^{2}=d_{2}^{2}+c_{2}^{2}+a_{2}^{2}+2d_{2}c_{2}\cos\alpha -2d_{2}a_{2}\cos\theta_{4}\\ \;\;\;\;\;\;\;\;\;\;\;\;\;\;\;\;\;\;\;\;\;\;\;\;-2c_{2}a_{2}(\cos\alpha \cos\theta_{4}+\sin\alpha \sin\theta_{4}) \end{array}$$

With the necessary arrangements, the objective function in the form of a polynomial is shaped with the construction parameters (unknown link lengths) and variables:

(6) $$\left(\frac{d_{2}^{2}+c_{2}^{2}+a_{2}^{2}-b_{2}^{2}}{2a_{2}c_{2}}\right)+\frac{d_{2}}{a_{2}}\cos\alpha -\frac{d_{2}}{c_{2}}\cos\theta_{4}-\cos\left(\theta_{4}-\alpha \right)=0$$

Equation (6) can be represented as the polynomial form as in Equation (7), in which the parameters were as in Equations (8) and (9):

(7) $$P_{0}f_{0}+P_{1}f_{1}+P_{2}f_{2}-F=0$$

where,

(8) $$P_{0}=\frac{d_{2}^{2}+c_{2}^{2}+a_{2}^{2}-b_{2}^{2}}{2a_{2}c_{2}},\;\;\;P_{1}=\frac{d_{2}}{a_{2}},\;\;\;P_{2}=-\frac{d_{2}}{c_{2}}$$

and,

(9) $$f_{0}=1,\;\;\;f_{1}=\cos\alpha ,\;\;\;f_{2}=\cos\theta_{4},\;\;\;F=\cos\left(\theta_{4}-\alpha \right)$$

In order to solve Equation (6), the values of terms *θ*_4_ and *α* should be given as precision points. As there exist only three unknowns in Equation (7) (*P*_0_*, P*_1_*, P*_2_) three equations, collected in Equation (10), are needed to reach the solution by three sets of precision points *α_i_ = f(θ*_4*i*_*), i =* 1, 2, 3:

(10) $$\begin{array}{l} P_{0}f_{0}^{1}+P_{1}f_{1}^{1}+P_{2}f_{2}^{1}-F_{1}=0\\ P_{0}f_{0}^{2}+P_{1}f_{1}^{2}+P_{2}f_{2}^{2}-F_{2}=0\\ P_{0}f_{0}^{3}+P_{1}f_{1}^{3}+P_{2}f_{2}^{3}-F_{3}=0 \end{array}$$

With three precision points, it is possible to obtain the construction parameters. The matrix form of Equation (10) is presented in Equation (11):

(11) $$\left[\begin{array}{ccc} f_{0}^{1} & f_{1}^{1} & f_{2}^{1}\\ f_{0}^{2} & f_{1}^{2} & f_{2}^{2}\\ f_{0}^{3} & f_{1}^{3} & f_{2}^{3} \end{array}\right]\left[\begin{array}{c} P_{0}\\ P_{1}\\ P_{2} \end{array}\right]=\left[\begin{array}{c} F_{1}\\ F_{2}\\ F_{3} \end{array}\right]$$

Construction parameters are as in Equation (12):

(12) $$\begin{array}{l} a_{2}=\frac{d_{2}}{P_{1}}\\ c_{2}=-\frac{d_{2}}{P_{2}}\\ b_{2}=\sqrt{d_{2}^{2}+c_{2}^{2}+a_{2}^{2}-2P_{0}a_{2}c_{2}} \end{array}$$

As a result, in order to define a four-bar mechanism that needs to mimic the biting action of the pharyngeal jaw of a moray eel, input–output relations at three different points and the distance between ground points (*d*_2_) must be given to this system of equations.

#### 3.1.2. Kinematic Synthesis of the Offset Slider–Crank Mechanism of the Pharyngeal Jaw

**Figure 11:**
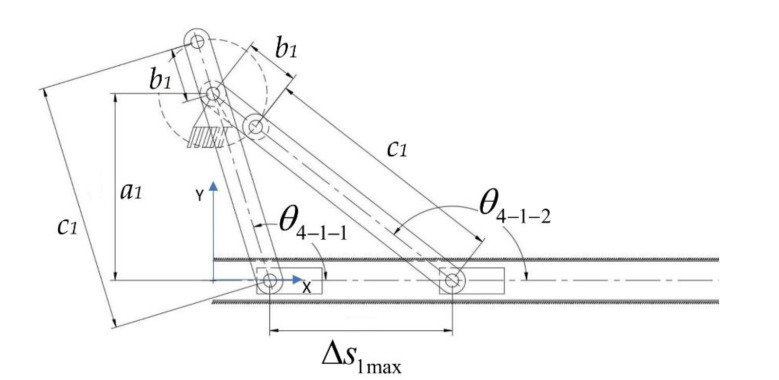
The Offset slider–crank mechanism poses at two dead-end points.

While the four-bar mechanism of the pharyngeal jaw is present for the biting action, another task of this jaw set is to travel from the throat to the outer oral jaw or vice-versa. As mentioned before, the motion of the pharyngeal jaw can be examined with two separate motions: pure rotation and pure translation. Translational motion is carried out by an offset slider–crank mechanism. This mechanism not only achieves the traveling (along with *s*_1_ in Figure 8b) but also carries and actuates the four-bar mechanism by the rotational motion of the rigidly connected *c*_1_ and *a*_2_ links (Figure 9). Here, two dead-end points are considered so as to synthesize the slider–crank mechanism (Figure 11).

From the trigonometric relations, construction parameters of the offset slider–crank mechanism are derived as following in Equation (13):

(13) $$\begin{array}{l} a_{1}=\Delta s_{1\max}\left[\frac{\sin\left(180-\theta_{4-1-1}\right)\sin\left(180-\theta_{4-1-2}\right)}{\sin\left(\theta_{4-1-2}-\theta_{4-1-1}\right)}\right]\\ b_{1}=\Delta s_{1\max}\left[\frac{\sin\left(180-\theta_{4-1-1}\right)-\sin\left(180-\theta_{4-1-2}\right)}{\sin\left(\theta_{4-1-2}-\theta_{4-1-1}\right)}\right]\\ c_{1}=\Delta s_{1\max}\left[\frac{\sin\left(180-\theta_{4-1}\right)+\sin\left(180-\theta_{4-1}\right)}{\sin\left(\theta_{4-1-2}-\theta_{4-1-1}\right)}\right] \end{array}$$

In this synthesis, procedure ∆*s*_1-max_, *θ*_4-1-1_*,* and *θ*_4-1-2_ are given to system equations according to the requirements of the jaw mechanism, which will be discussed in Section 4. Eventually, these requirements define the shape and limit of the offset slider–crank mechanism. Then, for constructing the overall shape of the pharyngeal jaw, biomimetic and functional design constraints for a combination of both mechanisms are considered in numerical works.

### 3.2. The Kinematic Synthesis of the Oral Jaw

The motion of the oral jaw is described as a pure rotation since its duty is to open and close the oral jaw within pre-defined time instants in order to catch the prey (Figure 10). The biomimetic motion is achieved by using the inverted slider–crank mechanism, where the sliding input is given to the system by a synthesized motion profile. Here, this mechanism is convenient to fulfill the requirements and increase the design variations in the work. Moreover, it is easy to place the input motion profile by means of a mechanical point of view.

Since the actuator on the system has a rotary output, a motion profile of the sliding motion is obtained by creating a motion profile with respect to requirements at each time instant.

#### The Kinematic Synthesis of the Inverted Slider–Crank Mechanism of the Oral Jaw

The oral jaw is the outer part of the mechanism, where the moray eel catches the prey with only a pure rotation motion as seen in Figure 6. In the previous sections, bio-constraints were defined for the motion of the oral jaw.

The biting action of the oral jaw is based on a rotation around a hinge, where the jaw achieves a closing and opening action. However, this rotation should work together sequentially with the pharyngeal jaw. Therefore, in the structural synthesis study, it was concluded that transferring the continuous rotational input motion to the slider–crank mechanism, so as to obtain a predefined oral jaw motion, was bound to the cam profile. In its simplest way, the system is built as an inverted slider–crank mechanism as in Figure 10.

As seen in Figure 10, there are some parameters that affect the efficiency of the resultant mechanism, thus, these parameters have some flexibility. For instance, we cannot conclude that the angle *ϕ* should be some sharp value due to the fact that it might be needing a different value to achieve the higher force transmission for biting, so the parameters *ϕ* and *β* are given to the system equations in the logical boundaries, which will be discussed in Section 4.2. Here, the analytic kinematic synthesis methodology is carried out as if those parameters are known and the variables are *s*_0_ and *φ*, which are the input stroke profile and the oral jaw angle, respectively.

Function generation kinematic synthesis starts with *x* and *y* components of closed-loop equations which can be visualized in Figure 10 and revealed in Equations (14) and (15):

(14) $$s_{0}\cos\phi +c_{0}\cos\gamma =e_{0}+d_{0}\cos\left(\varphi +\beta \right)$$

(15) $$a_{1}+s_{0}\sin\phi +c_{0}\sin\gamma =d_{0}\sin\left(\varphi +\beta \right)$$

In order to obtain a relation between *s*_0_ and *φ*, *γ* should be eliminated from the equations by adding the squares of Equations (16) and (17):

(16) $$c_{0}\cos\gamma =e_{0}+d_{0}\cos\left(\varphi +\beta \right)-s_{0}\cos\phi$$

(17) $$c_{0}\sin\gamma =d_{0}\sin\left(\varphi +\beta \right)-a_{1}-s_{0}\sin\phi$$

With the necessary arrangements, objective function in the form of a polynomial is shaped with the construction parameters (unknown link lengths) and variables:

(18) $$\begin{array}{l} \frac{d_{0}^{2}+e_{0}^{2}+a_{1}^{2}-c_{0}^{2}}{2}+d_{0}\left[e_{0}\cos\left(\varphi +\beta \right)-a_{1}\sin\left(\varphi +\beta \right)-s_{0}\cos\left(\varphi +\beta -\phi \right)\right]\\ \;\;\;\;\;\;\;\;\;\;\;\;\;\;\;\;\;\;\;\;\;\;\;\;\;\;\;\;\;\;\;\;\;\;\;\;\;\;\;\;\;\;\;\;\;\;\;\;\;\;\;\;\;\;\;\;\;\;\;-\left[\frac{-s_{0}^{2}}{2}+e_{0}s_{0}\cos\phi -a_{1}s_{0}\sin\phi \right]=0 \end{array}$$

Equation (18) can be represented as the polynomial form as in Equation (19), in which parameters were as in Equations (20) and (21):

(19) $$P_{0}f_{0}+P_{1}f_{1}-F=0$$

where,

(20) $$P_{0}=\frac{d_{0}^{2}+e_{0}^{2}+a_{1}^{2}-c_{0}^{2}}{2},\;\;\;P_{1}=d_{0}$$

and,

(21) $$\begin{array}{l} f_{0}=1\\ f_{1}=e_{0}\cos\left(\varphi +\beta \right)-a_{1}\sin\left(\varphi +\beta \right)-s_{0}\cos\left(\varphi +\beta -\phi \right)\\ F=\frac{-s_{0}^{2}}{2}+e_{0}s_{0}\cos\phi -a_{1}s_{0}\sin\phi \end{array}$$

In order to solve Equation (18), values of terms *φ* and *s*_0_ should be given as precision points. As there exist only two unknowns in Equation (19) (*P*_0_*, P*_1_) two equations, collected in Equation (22), are needed to reach the solution by two sets of precision points *φ_i_ = f(s*_0*i*_*), i =* 1, 2:

(22) $$\begin{array}{l} P_{0}f_{0}^{2}+P_{1}f_{1}^{1}-F_{1}=0\\ P_{0}f_{0}^{2}+P_{1}f_{1}^{2}-F_{2}=0 \end{array}$$

With two precision points, it is possible to get the construction parameters. The matrix form of Equation (22) is presented in Equation (23):

(23) $$\left[\begin{array}{cc} f_{0}^{1} & f_{1}^{1}\\ f_{0}^{2} & f_{1}^{2} \end{array}\right]\left[\begin{array}{c} P_{0}\\ P_{1} \end{array}\right]=\left[\begin{array}{c} F_{1}\\ F_{2} \end{array}\right]$$

Construction parameters are as in Equation (24):

(24) $$\begin{array}{l} d_{0}=P_{1}\\ c_{0}=\sqrt{d_{0}^{2}+e_{0}^{2}+a_{1}^{2}-2P_{0}} \end{array}$$

As a result, in order to define an inverted slider–crank mechanism that needs to mimic the biting action of the oral jaw of a moray eel, the input–output relation at two different points must be given to the system of equations.

## 4. Parameter Optimization

The main goal of this design is to create a single DOF mechanism that mimics the movements of moray eel jaws during hunting, which has three outputs in the view of kinematic representation. In general, after utilizing the kinematic synthesis procedures, the output position or orientation is optimized with various different techniques in order to reach the most accurate mechanism according to need. However, in this overall mechanism, there is no strict position or orientation needs at any instant, instead, relatively greater intervals are required such as at the specific instant minimum possible value of the jaw angle. For this mechanism, rather than positions and orientations, torque delivering at the time of when biting occurs is more crucial. Thus, it is focused on maximizing torque delivering at specific biting instants.

If the kinematic motion of the overall mechanism is examined, expected velocities of the mechanism parts are relatively slow. Together with this reason, although there are several methods available to analyze the force/torque relationship between the input and the output, instead of making a complex dynamic analysis, virtual work methodology is utilized without including the effects of the masses and inertias of the small links, which is negligible compared to the resultant biting forces.

In order to utilize the virtual work methodology in overall mechanism, position and velocity output depending on the crank and the prismatic joint input should be determined at every instant, thus, kinematic analysis in terms of position and velocity is a must.

### 4.1. Kinematic Analysis

After applying kinematic synthesis procedures to each individual mechanisms, kinematic analysis should be applied for simulating motions, utilizing virtual work method, and checking if the resultant mechanisms fit the constraints at different input angles.

#### 4.1.1. Kinematic Position Analysis of the Four-Bar Mechanism of the Pharyngeal Jaw

As in the synthesis case, position analysis also starts with the closed-loop equations as in Equations (1) and (2) and dismiss the angle *θ*_5_ as in Equations (3) and (4), eventually shaped Equations (25) and (26):

(25) $$\begin{array}{l} \frac{-b_{2}^{2}+d_{2}^{2}+a_{2}^{2}}{2}+c_{2}d_{2}\cos\alpha -a_{2}d_{2}\cos\theta_{4}-a_{2}c_{2}\cos\alpha \cos\theta_{4}\\ \;\;\;\;\;\;\;\;\;\;\;\;\;\;\;\;\;\;\;\;\;\;\;\;\;\;\;\;\;\;\;\;\;\;\;\;\;\;\;\;\;\;\;\;\;\;\;\;\;\;\;\;\;\;-a_{2}c_{2}\sin\alpha \sin\theta_{4}=0 \end{array}$$

(26) $$\begin{array}{l} \underset{M_{1}}{\underbrace{\left[a_{2}c_{2}\cos\theta_{4}-c_{2}d_{2}\right]}}\cos\alpha +\underset{M_{2}}{\underbrace{\left[a_{2}c_{2}\sin\theta_{4}\right]}}\sin\alpha \\ \;\;\;\;\;\;\;\;\;\;\;\;\;\;\;\;\;\;\;\;\;\;\;\;\;\;\;\;\;\;\;\;\;\;=\underset{M_{3}}{\underbrace{\left[\frac{-b_{2}^{2}+d_{2}^{2}+c_{2}^{2}+a_{2}^{2}}{2}-a_{2}d_{2}\cos\theta_{4}\right]}} \end{array}$$

From a half tangent formula, the procedure continues from Equation (27) to Equation (32):

(27) Let  2ψ=α

(28) $$\left(M_{1}\right)\cos2\psi +\left(M_{2}\right)\sin2\psi =M_{3}$$

(29) $$\frac{\left(M_{1}\right)\left[1-\tan^{2}\psi \right]}{1+\tan^{2}\psi }+\frac{\left(M_{2}\right)\left[2\tan\psi \right]}{1+\tan^{2}\psi }=M_{3}$$

(30) $$\underset{A}{\underbrace{\left(M_{1}+M_{3}\right)}}\tan^{2}\psi \underset{B}{\underbrace{-2M_{2}}}\tan\psi +\underset{C}{\underbrace{M_{3}-M_{1}}}=0$$

(31) $$\tan\psi =\frac{-B\pm \sqrt{B^{2}-4AC}}{2A}$$

(32) $$\psi =\tan^{-1}\left(\frac{-B\pm \sqrt{B^{2}-4AC}}{2A}\right)$$

With the following equations, we can determine the output angles at each input instants as:

(33) $$\alpha =2\tan^{-1}\left(\frac{-B\pm \sqrt{B^{2}-4AC}}{2A}\right)$$

(34) $$\theta_{5}=\mathrm{atan}2\left(c_{2}\sin\alpha -a_{2}\sin\theta_{4}\;,\;d_{2}+c_{2}\cos\alpha -a_{2}\cos\theta_{4}\right)$$

Time dependent varying values of input angle *θ*_4_ affect the outputs of *α* and *θ*_5_ as in Equations (33) and (34), which are used in a simulation process and in dynamic relations.

#### 4.1.2. Kinematic Position Analysis of the Offset Slider–Crank Mechanism of the Pharyngeal Jaw

Position analysis starts with the closed loop equations as in Equations (35) and (36) and dismiss the angle *θ*_3_ as in Equations (37) and (38) and continues with Equations (39) and (40):

(35) $$b_{1}\cos\theta_{2}+c_{1}\cos\theta_{3}=s_{1}$$

(36) $$a_{1}+b_{1}\sin\theta_{2}+c_{1}\sin\theta_{3}=0$$

(37) $$c_{1}\cos\theta_{3}=s_{1}-b_{1}\cos\theta_{2}$$

(38) $$c_{1}\sin\theta_{3}=-a_{1}-b_{1}\sin\theta_{2}$$

(39) $$c_{1}^{2}=s_{1}^{2}+a_{1}^{2}+b_{1}^{2}-2s_{1}b_{1}\cos\theta_{2}+2a_{1}b_{1}\sin\theta_{2}$$

(40) $$s_{1}^{2}-\left(2b_{1}\cos\theta_{2}\right)s_{1}+2a_{1}b_{1}\sin\theta_{2}+b_{1}^{2}+a_{1}^{2}-c_{1}^{2}=0$$

By letting discriminant as in Equation (41):

(41) $$\Delta ={\left(2b_{1}\cos\theta_{2}\right)}^{2}-4\left(2a_{1}b_{1}\sin\theta_{2}+b_{1}^{2}+a_{1}^{2}-c_{1}^{2}\right)$$

(42) $$s_{1}=\frac{2b_{1}\cos\theta_{2}\pm \sqrt{\Delta }}{2}$$

(43) $$\theta_{3}=\tan^{-1}\left(\frac{-a_{1}-b_{1}\sin\theta_{2}}{s_{1}-b_{1}\cos\theta_{2}}\right)$$

Time dependent varying values of input angle *θ*_2_ affect outputs of *s*_1_ and *θ*_3_ as in Equations (42) and (43), which are used in a simulation process and in dynamic relations.

#### 4.1.3. Kinematic Position Analysis of Inverted Slider–Crank Mechanism of Oral Jaw

As in the synthesis case, position analysis also starts with the closed-loop equations as in Equations (14) and (15) and dismiss the angle *γ* as in Equations (16) and (17), eventually shaped Equations (44) and (45):

(44) $$\begin{array}{l} \frac{-c_{0}^{2}+e_{0}^{2}+d_{0}^{2}+s_{0}^{2}+a_{1}^{2}}{2}+e_{0}d_{0}\cos\left(\varphi +\beta \right)-e_{0}s_{0}\cos\phi -d_{0}s_{0}\cos\phi \cos\left(\varphi +\beta \right)\\ \;\;\;\;\;\;\;\;\;\;\;\;\;\;\;\;\;\;\;\;\;\;\;\;\;\;\;\;\;\;\;\;\;\;\;\;\;\;-d_{0}s_{0}\sin\phi \sin\left(\varphi +\beta \right)-a_{1}d_{0}\sin\left(\varphi +\beta \right)+a_{1}s_{0}\phi =0 \end{array}$$

(45) $$\begin{array}{l} \left[e_{0}d_{0}-d_{0}s_{0}\cos\phi \right]\cos\left(\varphi +\beta \right)+\left[-a_{1}d_{0}-d_{0}s_{0}\sin\phi \right]\sin\left(\varphi +\beta \right)\\ \;\;\;\;\;\;\;\;\;\;\;\;\;\;\;\;\;\;\;\;\;\;\;\;\;=\left[\frac{-c_{0}^{2}+e_{0}^{2}+d_{0}^{2}+s_{0}^{2}+a_{1}^{2}}{2}+e_{0}s_{0}\cos\phi -a_{1}s_{0}\sin\phi \right] \end{array}$$

From a half tangent formula, the procedure continues from Equations (46) to (51):

(46) Let   2ψ=φ

(47) $$\left(L_{1}\right)\cos2\psi +\left(L_{2}\right)\sin2\psi =L_{3}$$

(48) $$\frac{\left(L_{1}\right)\left[1-\tan^{2}\psi \right]}{1+\tan^{2}\psi }+\frac{\left(L_{2}\right)\left[2\tan\psi \right]}{1+\tan^{2}\psi }=L_{3}$$

(49) $$\underset{A}{\underbrace{\left(L_{1}+L_{3}\right)}}\tan^{2}\psi \underset{B}{\underbrace{-2L_{2}}}\tan\psi +\underset{C}{\underbrace{L_{3}-L_{1}}}=0$$

(50) $$\tan\psi =\frac{-B\pm \sqrt{B^{2}-4AC}}{2A}$$

(51) $$\psi =\tan^{-1}\left(\frac{-B\pm \sqrt{B^{2}-4AC}}{2A}\right)$$

With the following equations, we can determine the output angles at each input instants as:

(52) $$\varphi =2\tan^{-1}\left(\frac{-B\pm \sqrt{B^{2}-4AC}}{2A}\right)-\beta$$

(53) $$\gamma =\mathrm{atan}2\left(d_{0}\sin\left(\alpha +\beta \right)-a_{1}-s_{0}\sin\phi \;,\;e_{0}+d_{0}\cos\left(\varphi +\beta \right)-s_{0}\cos\phi \right)$$

Time dependent varying values of input profile *s*_0_ affect outputs of *φ* and *γ* as in Equations (52) and (53), which are used in a simulation process and in dynamic relations.

#### 4.1.4. Kinematic Velocity Analysis of the Overall Pharyngeal Jaw Mechanism

In order to determine the velocities at each instant, kinematic closed-loop equations are used as follows:

Loop 1 in Equations (54) and (55):

(54) $$b_{1}\cos\theta_{2}+c_{1}\cos\theta_{3}+a_{2}\cos\left(\theta_{4-1}+\theta_{4-2}\right)+b_{2}\cos\theta_{5}=s_{1}+d_{2}+c_{2}\cos\left(\alpha_{1}+\alpha_{2}\right)$$

(55) $$a_{1}+b_{1}\sin\theta_{2}+c_{1}\sin\theta_{3}+a_{2}\sin\left(\theta_{4-1}+\theta_{4-2}\right)+b_{2}\sin\theta_{5}=c_{2}\sin\left(\alpha_{1}+\alpha_{2}\right)$$

Loop 2 in Equations (56) and (57):

(56) $$a_{2}\cos\left(\theta_{4-1}+\theta_{4-2}\right)+b_{2}\cos\theta_{5}=c_{2}\cos\left(\alpha_{1}+\alpha_{2}\right)+d_{2}$$

(57) $$a_{2}\sin\left(\theta_{4-1}+\theta_{4-2}\right)+b_{2}\sin\theta_{5}=c_{2}\sin\left(\alpha_{1}+\alpha_{2}\right)$$

Loop 1 Velocity from Equations (58) to (63):

(58) $$-b_{1}{\dot{\theta }}_{2}\sin\theta_{2}={\dot{s}}_{1}-c_{1}{\dot{\theta }}_{4-1}\sin\theta_{4-1}$$

(59) $$b_{1}{\dot{\theta }}_{2}\cos\theta_{2}=c_{1}{\dot{\theta }}_{4-1}\cos\theta_{4-1}$$

(60) $$\left[\begin{array}{c} -b_{1}\sin\theta_{2}\\ b_{1}\cos\theta_{2} \end{array}\right]\left\{{\dot{\theta }}_{2}\right\}=\left[\begin{array}{cc} 1 & -c_{1}\sin\theta_{4-1}\\ 0 & c_{1}\cos\theta_{4-1} \end{array}\right]\left[\begin{array}{c} {\dot{s}}_{1}\\ {\dot{\theta }}_{4-1} \end{array}\right]$$

(61) $${\dot{s}}_{1}=\frac{\left|\begin{array}{cc} -b_{1}{\dot{\theta }}_{2}\sin\theta_{2} & -c_{1}\sin\theta_{4-1}\\ b_{1}{\dot{\theta }}_{2}\cos\theta_{2} & c_{1}\cos\theta_{4-1} \end{array}\right|}{\left|\begin{array}{cc} 1 & -c_{1}\sin\theta_{4-1}\\ 0 & c_{1}\cos\theta_{4-1} \end{array}\right|}$$

(62) $${\dot{\theta }}_{4-1}=\frac{\left|\begin{array}{cc} 1 & -b_{1}{\dot{\theta }}_{2}\sin\theta_{2}\\ 0 & b_{1}{\dot{\theta }}_{2}\cos\theta_{2} \end{array}\right|}{\left|\begin{array}{cc} 1 & -c_{1}\sin\theta_{4-1}\\ 0 & c_{1}\cos\theta_{4-1} \end{array}\right|}$$

(63) $${\dot{\theta }}_{4-1}=\frac{{\dot{\theta }}_{2}b_{1}\cos\theta_{2}}{c_{1}\cos\theta_{4-1}}$$

Loop 2 Velocity from Equations (64) to (68):

(64) $$-a_{2}{\dot{\theta }}_{4}\sin\theta_{4}-b_{2}{\dot{\theta }}_{5}\sin\theta_{5}=-c_{2}\dot{\alpha }\sin\alpha$$

(65) $$a_{2}{\dot{\theta }}_{4}\cos\theta_{4}+b_{2}{\dot{\theta }}_{5}\cos\theta_{5}=c_{2}\dot{\alpha }\cos\alpha$$

(66) $$\left[\begin{array}{c} -a_{2}\sin\theta_{4}\\ a_{2}\cos\theta_{4} \end{array}\right]\left\{{\dot{\theta }}_{4}\right\}=\left[\begin{array}{cc} -b_{2}\sin\theta_{5} & -c_{2}\sin\alpha \\ b_{2}\cos\theta_{5} & c_{2}\cos\alpha \end{array}\right]\left[\begin{array}{c} {\dot{\theta }}_{5}\\ \dot{\alpha } \end{array}\right]$$

(67) $${\dot{\theta }}_{5}=\frac{\left|\begin{array}{cc} -a_{2}{\dot{\theta }}_{4}\sin\theta_{4} & -c_{2}\sin\alpha \\ a_{2}{\dot{\theta }}_{4}\cos\theta_{4} & c_{2}\cos\alpha \end{array}\right|}{\left|\begin{array}{cc} -b_{2}\sin\theta_{5} & -c_{2}\sin\alpha \\ b_{2}\cos\theta_{5} & c_{2}\cos\alpha \end{array}\right|}$$

(68) $$\dot{\alpha }=\frac{\left|\begin{array}{cc} -b_{2}\sin\theta_{5} & -a_{2}{\dot{\theta }}_{4}\sin\theta_{4}\\ b_{2}\cos\theta_{5} & a_{2}{\dot{\theta }}_{4}\cos\theta_{4} \end{array}\right|}{\left|\begin{array}{cc} -b_{2}\sin\theta_{5} & -c_{2}\sin\alpha \\ b_{2}\cos\theta_{5} & c_{2}\cos\alpha \end{array}\right|}$$

With the following Equation (69), we can determine the rotational velocity of the pharyngeal jaw at each input instant:

(69) $$\dot{\alpha }=\frac{b_{1}a_{2}{\dot{\theta }}_{2}\cos\theta_{2}\sin\left(\theta_{4}-\theta_{5}\right)}{c_{1}c_{2}\cos\theta_{4-1}\sin\left(\alpha -\theta_{5}\right)}$$

Time dependent varying position and velocity values of input angle *θ*_2_ affect rotational velocity of the pharyngeal jaw ($\dot{\alpha }$) in Equation (69), which will be used in dynamic analysis.

#### 4.1.5. Kinematic Velocity Analysis of the Overall Oral Jaw Mechanism

In order to determine the velocities at each instant, kinematic closed-loop equations, Equations (70) and (71), were used as follows from Equations (72) to (74):

(70) $${\dot{s}}_{0}\cos\phi =c_{0}\dot{\gamma }\sin\gamma -d_{0}\dot{\varphi }\sin\left(\varphi +\beta \right)$$

(71) $${\dot{s}}_{0}\sin\phi =-c_{0}\dot{\gamma }\sin\gamma +d_{0}\dot{\varphi }\cos\left(\varphi +\beta \right)$$

(72) $$\left[\begin{array}{c} \cos\phi \\ \sin\phi \end{array}\right]\left\{{\dot{s}}_{0}\right\}=\left[\begin{array}{cc} c_{0}\sin\gamma & -d_{0}\sin\left(\varphi +\beta \right)\\ -c_{0}\cos\gamma & d_{0}\cos\left(\varphi +\beta \right) \end{array}\right]\left[\begin{array}{c} \dot{\gamma }\\ \dot{\varphi } \end{array}\right]$$

(73) $$\dot{\varphi }=\frac{\left|\begin{array}{cc} c_{0}\sin\gamma & {\dot{s}}_{0}\cos\phi \\ -c_{0}\cos\gamma & {\dot{s}}_{0}\sin\phi \end{array}\right|}{\left|\begin{array}{cc} c_{0}\sin\gamma & -d_{0}\sin\left(\varphi +\beta \right)\\ -c_{0}\cos\gamma & d_{0}\cos\left(\varphi +\beta \right) \end{array}\right|}$$

(74) $$\dot{\varphi }=\frac{{\dot{s}}_{0}\cos\left(\gamma -\phi \right)}{d_{0}\sin\left(\gamma -\varphi -\beta \right)}$$

Time dependent varying position and velocity values of input profile *s*_0_ affect rotational velocity of the oral jaw ($\dot{\varphi }$) in Equation (74), which will be used in dynamic analysis.

### 4.2. Input Sets of Mechanisms

Since the main concern here is to mimic the desired action of jaws, kinematic position error is not critical, therefore, it is not needed to be so precise in terms of position, and, therefore, the motion characteristic is more important. By concerning the anti-parallel four-bar mechanism that slides as a whole, which makes the biting action of the pharyngeal jaw, the output jaw angle should have specific values at certain moments such as when biting takes place or at a time when the jaw is fully opened. At the same time, the oral jaw and the slider should also be at predefined positions, which were given as bio-constraints in Section 2. However, corresponding input angles cannot be specified sharply in the first place, since input has the flexibility of being in a different range. For instance, the input range of the four-bar mechanism (*θ*_4_) of the pharyngeal jaw can be 30° as well as 90°, which directly affects the slider–crank mechanism. Here, what eventually matters is specified output angle.

For these reasons, it was decided that we should create an algorithm by using a computing program in order to utilize different kinematic synthesis by changing the input ranges as well as the additional parameters. These additional parameters are *α_offset_* for the four-bar mechanism of the pharyngeal jaw (Figure 8a), *θ*_4-2_ for the slider–crank mechanism of the pharyngeal jaw (Figure 9), and *ϕ* and *β* for the inverted slider–crank mechanism of the oral jaw (Figure 10). Firstly, input sets of all mechanisms should be created in a logical manner.

#### 4.2.1. Input Sets of the Four-Bar Mechanism of the Pharyngeal Jaw

By considering the structural synthesis of combined mechanisms, while the slider–crank mechanism part carries the whole pharyngeal jaw by translating it out to the end of the mouth, it has a motion on *+x* direction (towards the end of the mouth) as the angle between *+x* and link *c*_1_ gets bigger (Figure 9). As the jaw slides out to the end of the mouth, the biting action should take place, which means that the jaw angle (*α-α_offset_*) should get smaller, close to zero degrees. However, the initial value of the input angle of the sliding anti-parallel four-bar mechanism (*θ*_4_) can be extremely flexible, therefore, its range of motion is limited to between 270° and 360°. Moreover, the difference between the initial and the final angle of *θ*_4_ is chosen between 90° and 30°. With this information, an array of set of initial *θ*_4_ and an array of set of final *θ*_4_ are created with a step size of 5°. As mentioned in the kinematic synthesis part, the sliding four-bar mechanism needs three precision points to be constructed, where the remaining *θ*_4_ is defined with linear spacing. A numerical example of the first 8 index of *θ*_4_ array can be seen in Table 2.

The output set of the sliding four-bar mechanism should not change whereas the input set changes. When the input is on the state of initial *θ*_4_, the pharyngeal jaw should be open, which is considered to be 45°, with the jaw angle as an open state. For the next step, when the input is on the state of mean *θ*_4_, the pharyngeal jaw should be approximately half open, which is considered to be 20°, with the jaw angle as a medium state. Finally, when the input is on the state of final *θ*_4_, the pharyngeal jaw should be fully closed (biting state), which is considered to be 3°, with the jaw angle as a closed state. However, as seen from the mechanism synthesis (Figure 9), there is also a platform at the end of the considered jaw, which directly affects the mechanism synthesis results, thus, its shape is flexible as well. For this reason, the angle in the platform (*α_offset_*) is also considered to be variable, therefore, multiple αoffset angle values are created with the offset from 60° to 160° with a step size of 1°.

For each αoffset and *θ*_4_ difference values, 1911 different input sets are created and all of them are ready to be used in the synthesis study of the four-bar mechanism on the slider, which is eventually used in the biting action.

#### 4.2.2. Input Sets of the Slider–Crank Mechanism of the Pharyngeal Jaw

In order to size the overall mechanism that mimics the average moray eel, the slider travel is chosen as 140 mm, which directly carries the pharyngeal jaw mechanism from throat to the end of the mouth. As seen in Figure 9, there is a fixed offset angle (*θ*_4-2_), which creates a platform on the mechanism. This fixed angle creates flexibility on the design, which should not be chosen randomly because of the effect on the overall system. Therefore, in the algorithm, another loop is carried out in which the slider offset angle (*θ*_4-2_) is changed from 91° to 225° with a step size of 1°. Together with the previous selections, the input set increases to 9164 different combinations.

For each usable sliding four-bar mechanism, which are determined in previous section, with initial *θ*_4_ angle with specified *θ*_4-2_ offset, analytical kinematic synthesis with two positions is carried out for offset slider–crank mechanism. As a result, if the construction parameters (link lengths of the offset slider–crank mechanism) is real and positive, it is possible to conclude that offset the slider–crank mechanism is suitable to work with the sliding four-bar mechanism together without any constraint.

#### 4.2.3. Input Sets of the Inverted Slider–Crank Mechanism of the Oral Jaw

By considering Figure 10, input of the oral jaw is actually a prismatic joint (*s*_0_), whose motion can be given to the system as an input depending on the crank position by using a cam profile that drives the instantaneous output jaw angle, which is rotated to open or close oral jaw itself at every instant in one full turn of the crank. Eventually, the cam profile is actually extracted as a need to give the required motion for the prismatic joint in the oral jaw. Here, the flexibilities are on the direction of the input profile (*ϕ*) and output jaw platform (*β*), thus, these parameters should be multiple in logical boundaries. Moreover, the range and initial value of the input profile *s*_0_ affects the shape and the force transmission of the resultant mechanism, as in the case of the input angle of the sliding anti-parallel four-bar mechanism (Section 4.2.1).

In logical boundaries, angle *ϕ* is from −50° to 50°, while angle *β* should change from 60° to 150° with 5° step sizes. The input profile should also be within predefined values, which are from 20 mm to 50 mm with an initial value of 20 mm. For the different values of each parameter, a kinematic synthesis procedure was applied.

### 4.3. Multiple Synthesis Algorithm

Having flexibility on the parameters is a challenge for the design procedure due to the fact that it affects resultant actions dramatically. For this reason, the input–output relations were created within the logical boundaries so that the multiple results were concluded. Among these, the best result was chosen to be the one that has the highest output to input torque ratio at critical moments, which is at the fully closed position in order to increase the biting force, so that the smallest actuator can serve as the best torque transformer. Moreover, it is important to note that there are some functional kinematic constraints that depend on the motion characteristic to be followed by the overall mechanism in order to mimic the motion.

**Figure 12:**
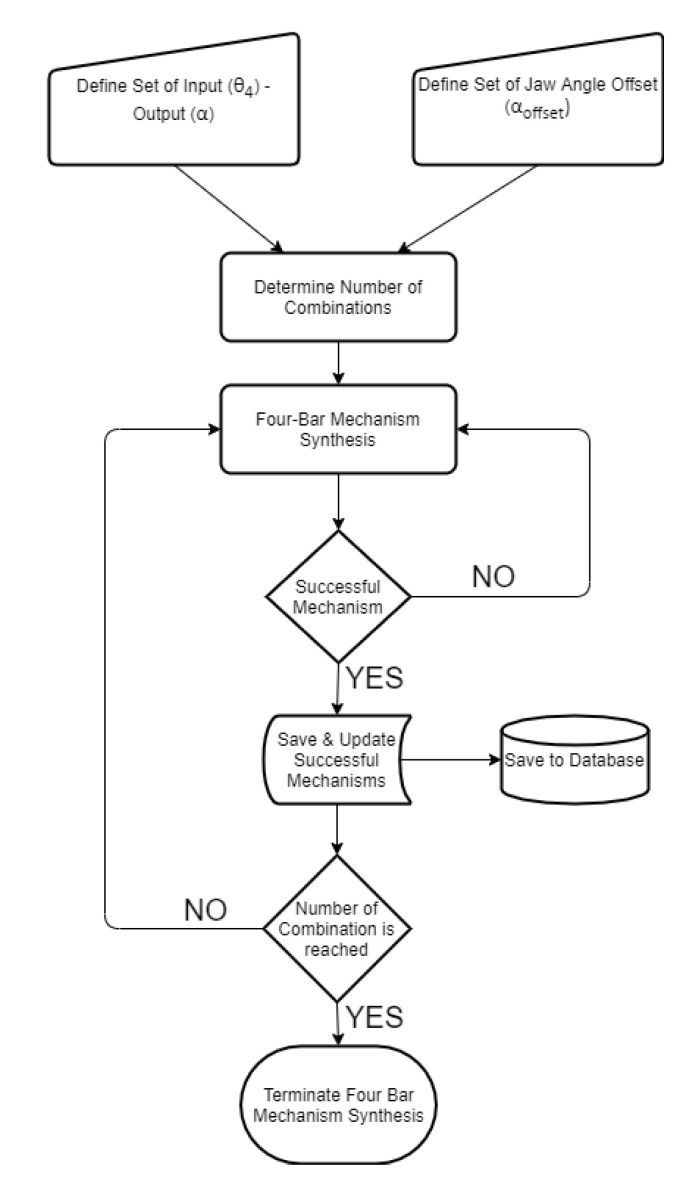
Algorithm for designing the four-bar mechanism at the pharyngeal jaw.

Defining input sets as discussed in previous sections, the flow of the multiple synthesis algorithm is based on a predefined logic. In this regard, a novel algorithm which is based on the kinematic synthesis is created in order to define the input–output set and the starting positions. The created algorithm, which worked in a MATLAB environment, starts with defining the input–output and the jaw angle sets of the sliding four-bar mechanism according to the fundamental needs of the biting action of the pharyngeal jaw. The needed equations for the synthesis study of the sliding four-bar mechanism are derived in Equation (12). As an input set changes with a predefined logic, a loop for determining the construction parameters (four-bar mechanism link lengths) is executed. For each input set, analytically calculated resultant link lengths are compared to zero, since a negative link length is not possible. If all link lengths are larger than zero, then it is possible to conclude that the resultant four-bar mechanism is usable without considering main constraints and design goals. As a result of the multiple synthesis for each input sets, besides link lengths, initial and final input angles (*θ*_4_) and a jaw offset angle (*α_offset_*) at the action of biting (slider is at the positive dead end) that meet predefined criteria are saved in the memory to be used later (Figure 12).

**Figure 13:**
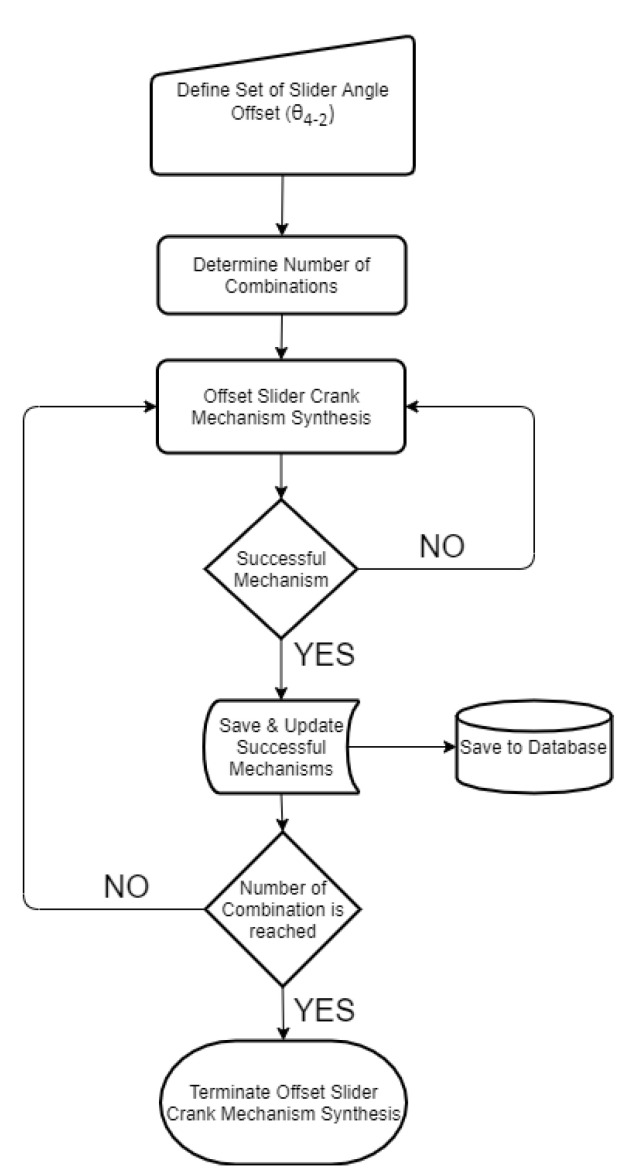
Algorithm for designing the offset slider–crank mechanism at the pharyngeal jaw.

The offset slider–crank mechanism also has a predefined fixed parameter to be used in the synthesizing procedure (*θ*_4-2_). However, according to the requirements in output, this parameter has flexibility as well. For this reason, an interval is defined, where they are used in synthesizing the offset slider–crank mechanism for each step. By combining the successful outputs of the four-bar mechanisms and a flexible parameter, the synthesis procedure is applied for each step and the ones that meet predefined criteria are saved in the memory as in the previous case (Figure 13).

At this step, it is reached some successful four-bar mechanisms and offset slider–crank mechanisms, which meet their criteria separately. The combination of these mechanisms should meet newer combined criteria, which are the main requirements for mimicking the pharyngeal jaw. The whole system should achieve the specific motion functional constraints that are formed by following:

- At the negative dead end of the slider–crank mechanism, the pharyngeal jaw should be open.
- From the negative dead end to the near-positive dead end, the pharyngeal jaw should be open during travel.
- At the positive dead end, the pharyngeal jaw should be closed (action of biting).
- From the positive dead end to the negative dead end, the pharyngeal jaw should be closed during travel.

**Figure 14:**
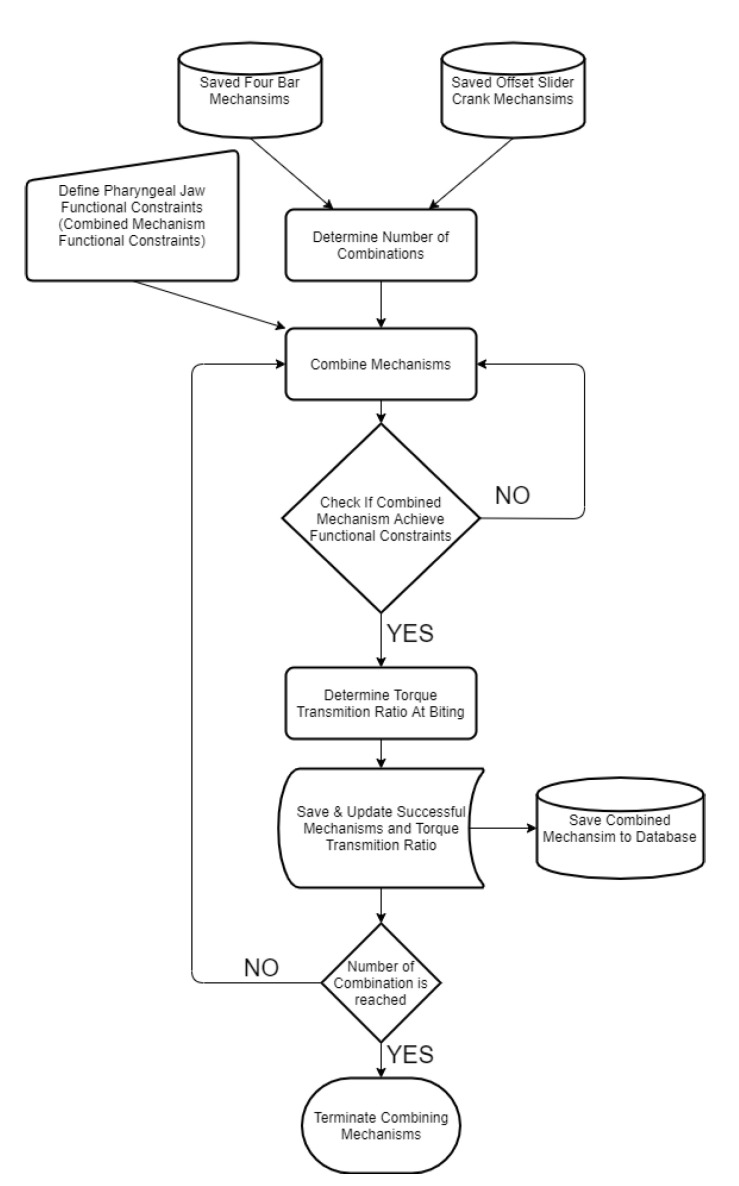
Algorithm for combining the four-bar mechanism and the offset slider–crank mechanism to get a functional pharyngeal jaw.

In order to achieve the above specific motion combinations, motion analysis of each created mechanism was carried out for a full revolution of the main input crank. At specified instants, the angles were compared to where they should occur. The first instant is the moment when slider is on the middle of travel from positive end to negative end (pulling the prey action), at which the pharyngeal jaw angle should be smaller than 5°. This means that the sliding four bar should be close to the pharyngeal jaw while being pulled into the esophagus. The second instant is the moment when slider is again in the middle of travel from the negative end to the positive end (catching the prey action), at which the pharyngeal jaw angle should be greater than 25°. This means that the sliding four bar should open the pharyngeal jaw while being pushed to the mouth’s end. This algorithm eliminates impractical mechanisms which are not needed to be optimized for the purpose of mimicking the moray eel’s pharyngeal jaw (Figure 14).

The next step is to design the oral jaw by considering the design constraints. As discussed in the synthesis procedure, two different precision points are required in order to define the mechanism. Here, the two dead ends of the input slider profile are given as flexible and the corresponding outputs of the main jaw mechanism are fully opened and fully closed. Motion in between these two ends is extracted according to the one cycle movement in one full turn, where it is fulfilled by the cam profile.

**Figure 15:**
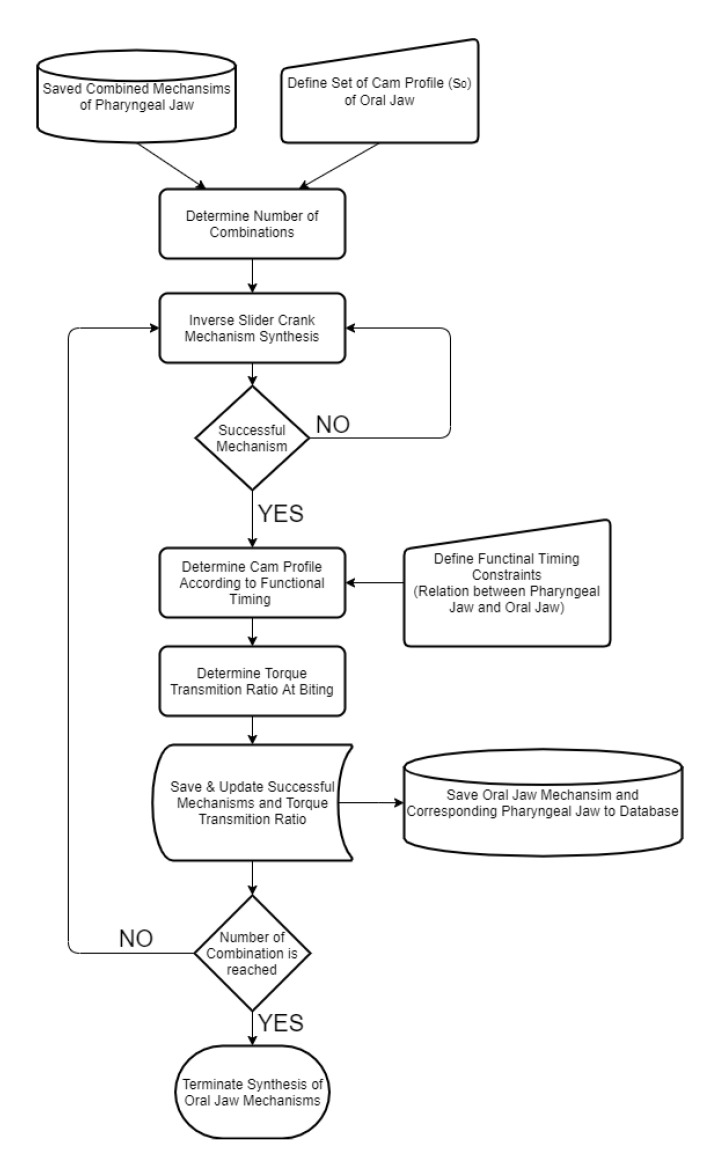
Algorithm for designing the inverse slider–crank mechanism at the oral jaw.

After utilizing the kinematic synthesis procedure for the oral jaw mechanism, according to the functional timing of the overall movement cycle, medium movements are extracted as defined by the cam profile. Functional timing of the overall movement cycle was approximately predicted in Figure 4. The resultant mechanisms that meet the criteria with created cam profiles were saved in the memory along with their important parameters (Figure 15).

In order to have an overall mechanism that completely mimics the motion of the two jaws of the moray eel, successful mechanisms should achieve the defined criteria. Eventually, it is possible to reach many distinct mechanisms, where optimization is needed.

### 4.4. Optimization of Mechanisms

With an excessive amount of resultant possible mechanisms, the most effective one should be taken into account. The effectiveness depends on certain parameters which affect overall efficiency, quality, and usability. In this design, the first criteria is chosen as force/torque transmitting from the actuator to both jaws, the oral and the pharyngeal jaw, when the biting action occurs, separately. From the input link to the output jaws, there are various links and connections to transform the desired motions. As mentioned in earlier sections, relative to required forces in the outputs, all of the masses and inertias can be neglected in order to simplify the force/torque analysis. In this work, a virtual work method is used for calculating the relationship between the input and the output torques.

#### 4.4.1. The Optimization of the Pharyngeal Jaw

For the pharyngeal jaw, the first thing one must consider is the torque transmission in the anti-parallel four-bar mechanism, which plays a vital role in biting prey and pulling it through the esophagus. For this reason, this biting torque should be as high as possible in order to avoid the prey fleeing. With a unit applied, the torque from the input actuator, and the torque at the pharyngeal jaw can be calculated as in Equations (75) and (76):

(75) $$\tau_{0}\delta \theta_{2}=\tau_{pharyngeal\;jaw}\delta \alpha$$

(76) $$\tau_{pharyngeal\;jaw}=\frac{\tau_{0}{\dot{\theta }}_{2}}{\dot{\alpha }}$$

By using the result from the velocity analysis of the pharyngeal jaw, kinematic Equation (76) is combined with Equation (69), that shapes Equation (77), in order to determine torque transmission:

(77) $$\tau_{pharyngeal\;jaw}=\frac{\tau_{0}\left[c_{1}c_{2}\cos\theta_{4-1}\sin\left(\alpha -\theta_{5}\right)\right]}{b_{1}a_{2}\cos\theta_{2}\sin\left(\theta_{4}-\theta_{5}\right)}$$

Since the main objective is to keep the *τ_pharyngeal jaw_* to a maximum at the time when the biting occurs, this equation should be applied to all resultant possible mechanisms with a unit of *τ*_0_.

#### 4.4.2. Optimization of the Oral Jaw

For the oral jaw, the main actuator creates the required torque that is then transformed to a linear force along with the slider. With the neglected masses and inertias compared to the desirable forces and torques, the torque transmission from the actuator to the teeth can be determined with a virtual work method.

**Figure 16:**
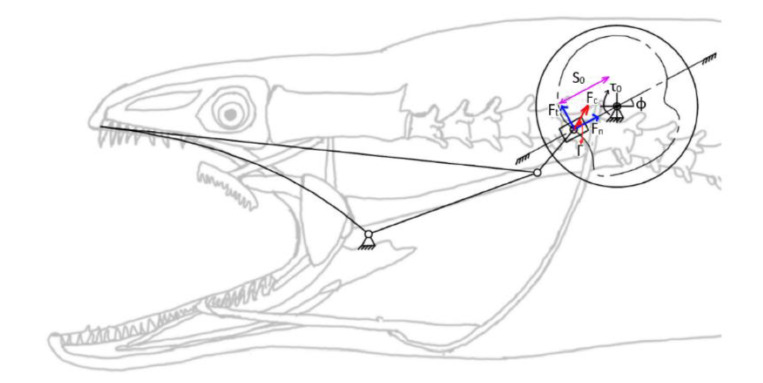
Force vectors of the cam profile on the oral jaw mechanism.

The force on the slider depends on the cam profile as seen in Figure 16 with the unit input torque (*τ*_0_) and can be calculated with Equation (78):

(78) $$F_{n}=\frac{F_{t}}{\tan\Gamma }=\frac{\tau_{0}}{s_{0}\tan\Gamma }$$

where Γ can be determined with vector operations.

Tangential force drives the slider linearly, eventually it results in squeezing the oral jaw of the moray eel as in Equations (79) and (80):

(79) $$F_{t}\delta s_{0}=\tau_{oral\;jaw}\delta \varphi$$

(80) $$\tau_{oral\;jaw}=\frac{\tau_{0}d_{0}\sin\left(\gamma -\varphi -\beta \right)}{s_{0}\cos\left(\gamma -\phi \right)\tan\Gamma }$$

The main objective is to keep the *τ_oral jaw_* to a maximum at the time that the biting occurs, this equation should be applied to all resultant possible mechanisms with a unit of *τ*_0_.

## 5. Results and Discussions

In order to reach the ultimate goal of this work, all of the needed algorithms and analytic equations are derived in the previous sections. Since the procedure consists of different methodologies, it can be summarized with its application flow as follows:

- Define the input sets of the four-bar mechanism of the pharyngeal jaw (*θ*_4_)
- Define the input sets of the four-bar mechanism output platform angle (*α_offset_*)
- Define the four-bar mechanism scale according to the requirement for size (*d*_2_)
- Utilize the Equations (11) and (12) for each input set in order to get the construction parameters of the four-bar mechanism of the pharyngeal jaw (*a*_2_*, b*_2_*, c*_2_)
- Define the pharyngeal jaw’s sliding travel (*∆s*_1_)
- Define the input sets of the slider offset angle (*θ*_4-2_)
- Utilize Equation (13) for each input set in order to get the construction parameters of the offset slider–crank mechanism of the pharyngeal jaw (*a*_1_*, b*_1_*, c*_1_)
- Combine both pharyngeal jaw mechanisms that follow the functional constraints and then identify the one with the highest torque transmitting ratio at the moment of the pharyngeal jaw biting
- Define the input sets of the inverted slider–crank mechanism of the oral jaw (*s*_0_)
- Define the input sets of the direction of the slider profile (*ϕ*)
- Define the input sets of the output oral jaw platform (*β*)
- Define the inverted slider–crank mechanism scale according to the requirement for the size (*e*_0_)
- Utilize Equations (23) and (24) for each input set in order to get the construction parameters of the inverted slider–crank mechanism of the oral jaw (*c*_0_, *d*_0_*,* and the resultant *s*_0_ interval)
- Combine all of the possible functional oral jaw mechanisms with the specified pharyngeal jaw and then identify the one with the highest torque transmitting ratio at the moment of oral jaw biting

After utilizing the procedure above, the resultant mechanism is determined in terms of millimeters and degrees as shown in Table 3.

**Figure 17:**
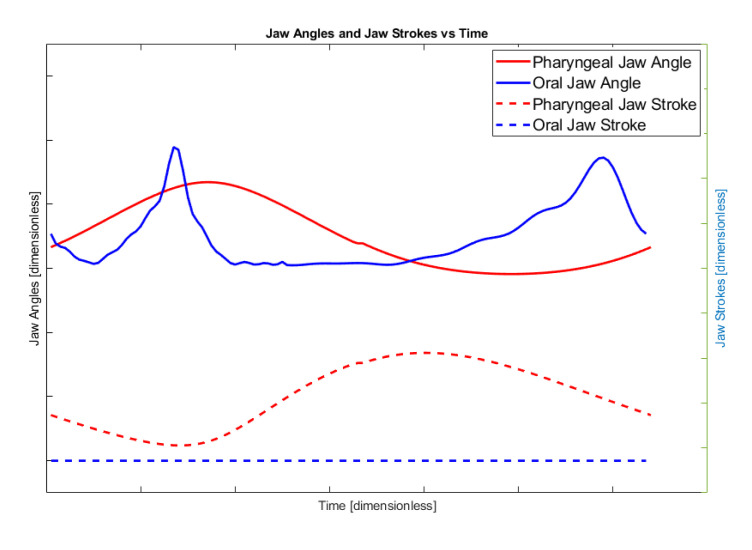
Resultant position and angle for both jaws.

In terms of kinematics, the resultant mechanism gives an output curve in one full rotation of the main actuator crank as seen in Figure 17. In Figure 4, the desired biting sequence is illustrated. Timing in the orientations of the jaws in Figure 4 was predicted in order to ensure harmony between the two jaws. A comparison between Figure 4 and Figure 7 show that there exists a difference in the angles of the jaws. The main reason for this difference is one of the design goals of the optimization study. The proposed mechanical structure was generated with both kinematic and dynamic constraints. The constraint, which causes profile shifting in the biting sequence, is the maximization of the force/torque rate. In order to maximize the transferred torque through the pharyngeal jaw, it is necessary to manipulate the derivative of the position equation of the pharyngeal jaw. Therefore, optimization of the torque delivery through the pharyngeal jaw is dependent on the velocity equations (Equation (76)). This relationship between the optimization study and the dynamic behavior of the pharyngeal jaw causes the difference in the biting sequence of this jaw.

**Figure 18:**
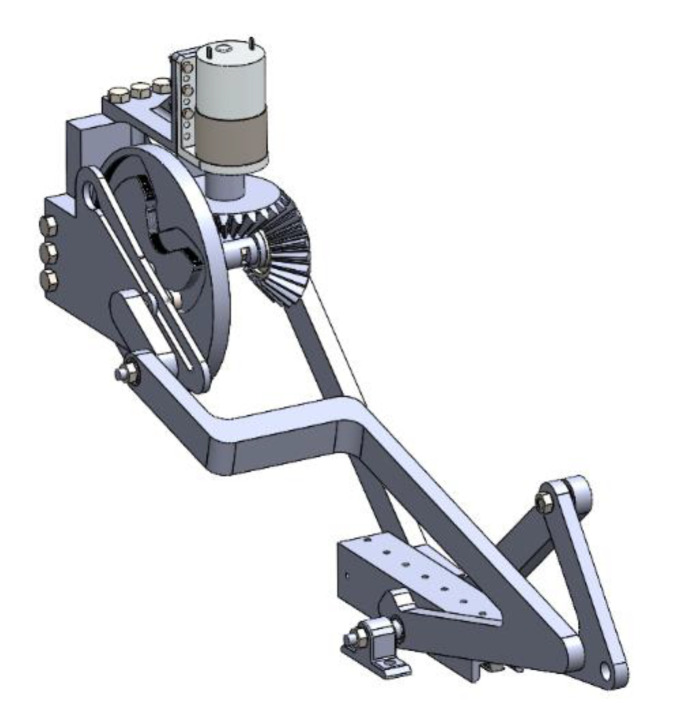
Isometric view of the concept design of the proposed mechanism.

The resultant mechanism is designed roughly in SolidWorks, as seen in Figure 18, in order to simulate the resultant movement as well as perform the needed dynamic analysis at critical instants with a different computer program.

**Figure 19:**
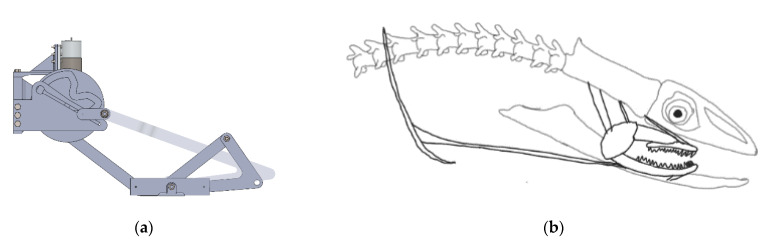
The pharyngeal jaw at biting instant: (**a**) as the mechanism; (**b**) as the moray illustration.

In a MATLAB environment, all of the optimization and kinematic synthesis procedures are utilized. The two critical biting instants are determined in terms of variables. These variables for the pharyngeal jaw are shown in Table 4. At biting instance, the torque ratio is revealed as being 7.28 by using Equation (77). At this critical instant, the kinematic design in SolidWorks can also be seen in Figure 19a with a corresponding position of moray illustration in Figure 19b.

In this section, the control of the result of the mathematical model is carried out by using the Multi Body Dynamics (MBD) simulation method. In this method, the simulation CAD model was created by using construction parameters and variables. The model does not include manufacturing details or components but geometrical details for analysis.

**Figure 20:**
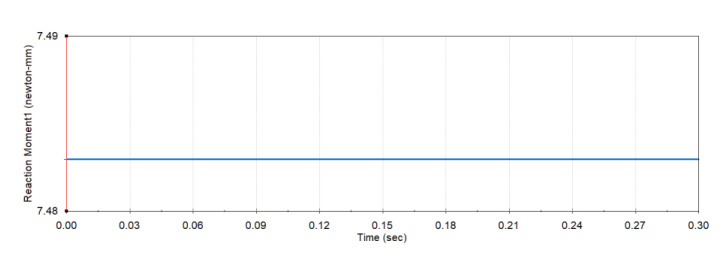
Torque transmitting ratio determined with MBD.

The MBD simulation model of the proposed mechanism was integrated into simulation environments with boundary conditions and initial conditions. These conditions ensure the exact data of the transferred torque through the jaw at that instant time. In order to obtain this data, the joint of the pharyngeal jaw was locked at the biting time and unit vector torque 1 [N/mm] was applied through the input link. As a result of the simulation, the reaction torque was measured on the joint of the jaw. The simulation results as a reaction torque are shown in Figure 20. Due to the applied torque magnitude, the output torque is equal to the torque conversion ratio, derived from Equation (77). It was determined at 7.48, which deviates from the analytic result by 2.75%.

**Figure 21:**
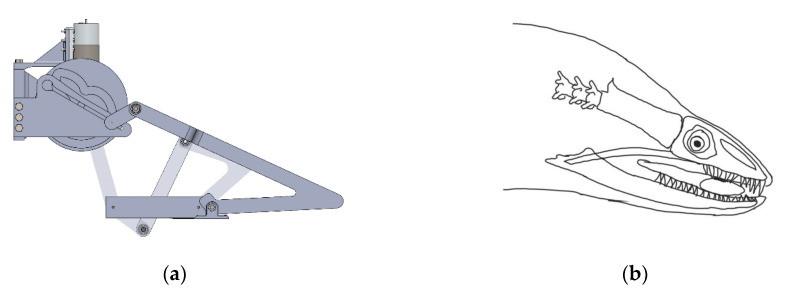
The oral jaw at biting instant: (**a**) as the mechanism; (**b**) as the moray illustration.

Similarly, other critical biting instants for the oral jaw were determined in terms of variables as well and can be seen in Table 5 with its SolidWorks kinematic design in Figure 21a with corresponding position of moray illustration in Figure 21b.

The powerful nature behind the biomimetic designs can inspire similar designs. The utilized procedure stems from a biomimetic design with the aim of reducing the task DOF. This procedure can be applied to different kinds of tasks that are mechanically connected to each other. In this work, the optimization parameter was chosen as the torque transferring ratio. By following similar multiple analytical kinematic synthesis for input–output flexibility, not only is the torque transferring ratio chosen as the optimization parameter but it can also be a kinematic position or an orientation error, by using existing optimization methodologies. The main idea here is to have a procedure that can be followed in order to get a combined mechanism that gives multiple outputs with a single degree of freedom. For similar repeating tasks with such designs, it is possible to reduce the existing control and timing complexity and increase the safety. By considering the simulations with different tools, it is shown that the possibility of such designs can be adapted easily and can verify the techniques that are used. This kind of procedure can be applied especially to automation systems that repeat the movements in which more DOF was already used and where algorithms are utilized. With a quicker kinematic design and embedded optimization, the overall cost can be reduced due to there being no need for more actuators, sensors, and complex control algorithms.

## 6. Conclusions and Future Works

In this work, a mechanism that mimics the double jaw apparatus of the moray eel was designed and optimized with a lower DOF than task DOF. A mechanism with a single actuator point drives the resultant mechanism and forms the kinematic movements. In the kinematic synthesis procedure, the bio-constraints were defined and, depending on those constraints, some construction parameters were forced into logical intervals. A structural design procedure was utilized in order to reach the ultimate goal and the dimensioning part was carried out with multiple kinematic synthesis algorithms due to the flexibility in terms of inputs and constraints. The procedure started with the pharyngeal jaw, which slides and rotates simultaneously. Depending on the resultant construction parameters of the pharyngeal jaw, a similar approach was utilized for the oral jaw. As a result of the algorithm, many different mechanisms were created, among which the most torque delivering device at critical instants was chosen. The procedure was explained in detail including bio-constraints, structural synthesis, kinematic synthesis, and optimization. In the end, the resultant mechanism was roughly designed in SolidWorks in order to visualize the movements.

In future work, the mechanical design will be improved and the manufacturing process will take place. The plan is to carry out the validation procedure in terms of kinetics and kinematics. A kinematic synthesis and optimization procedure is handled with a software that is developed in a MATLAB environment. However, since there are many parameters that should be specified by the user, it is not easy to hard-code using the synthesis and optimization program once the different requirements have arisen. Since the system can be used in a different automation process or in a similar project, a user-friendly graphical user interface is planned to be utilized so that the user can alter the parameters easily. Besides the torque transmitting option, the kinematic movement optimization algorithms will also be applied to this interface so that the user can decide which parameter would be optimized according to their needs. Moreover, a visual simulation will be added to this interface so that the user will be able to see the results easily, apart from just seeing the numerical values. In the end, this kind of design process could be utilized by anybody who needs such a design.

## Figures and Tables

**Table 1:** Approximation of the different poses of the oral jaw and the pharyngeal jaw during hunting.

Oral Jaw		Pharyngeal Jaw	
Natural Pose	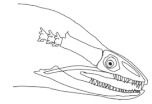	Slider to Backward, Jaw Closed–Opening	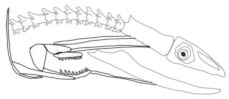
Opening for Biting	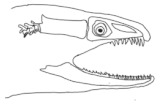	Dependent on Kinematic	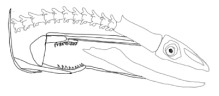
Max Jaw Angle	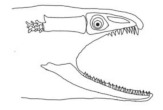	Slider at Back, Jaw Open	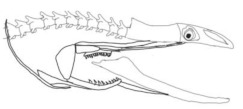
Closing for Biting	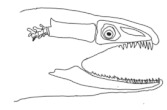	Slider to Forward, Jaw Open	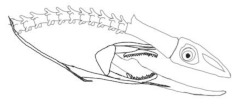
Biting	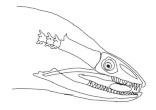	Slider to Forward Jaw Open	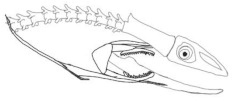
Starting Release	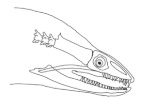	Slider to Forward, Jaw Open–Closing	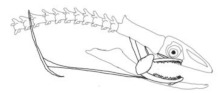
Released	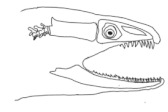	Slider at Forward, Jaw Closed	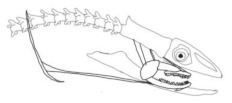
Back to Natural Pose	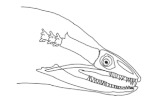	Slider to Backward, Jaw Closed	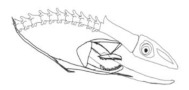
Natural Pose	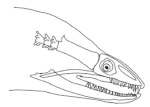	Slider to Backward, Jaw Closed–Opening	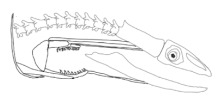

**Table 2:** Numerical example of the first 8 index of *θ*_4_.

Array Index	1	2	3	4	5	6	7	8
**Difference**	90°	85°	85°	80°	80°	80°	75°	75°
***θ*_4_ (initial)**	270°	270°	275°	270°	275°	280°	270°	275°
***θ*_4_ (mean)**	315°	312.5°	317.5°	310°	315°	320°	307.5°	312.5°
***θ*_4_ (final)**	360°	355°	360°	350°	355°	360°	345°	350°

**Table 3:** Resultant construction parameters.

Parameter	Value	Parameter	Value	Parameter	Value
*a*_1_	143.7	*b*_1_	41.6	*d*_2_	120
*s*_0_	22–92	*c*_1_	191.8	*ø*	−30°
*c*_0_	38.9	*a*_2_	52.6	*β*	125°
*d*_0_	174.7	*b*_2_	136.9	*θ*_4-2_	218°
*e*_0_	183.9	*c*_2_	94.1	*α_offset_*	60°

**Table 4:** Variables at the instant of pharyngeal jaw biting.

**Parameter**	*θ*_2_	*θ*_3_	*θ*_4_	*θ*_5_	*α*
**Value**	−45.82°	−36.32°	1.60°	37.27°	63.76°

**Table 5:** Variables at the instant of oral jaw biting.

**Parameter**	*θ*_2_	*γ*	*s*_0_	*φ + β*
**Value**	56.33°	36.80°	55	127.00°

## Data Availability

Not applicable.
